# In Vitro microRNA Expression Profile Alterations under CDK4/6 Therapy in Breast Cancer

**DOI:** 10.3390/biomedicines11102705

**Published:** 2023-10-05

**Authors:** Jasmin Asberger, Kai Berner, Anna Bicker, Marius Metz, Markus Jäger, Daniela Weiß, Clemens Kreutz, Ingolf Juhasz-Böss, Sebastian Mayer, Isabell Ge, Thalia Erbes

**Affiliations:** 1 Department of Obstetrics and Gynecology, Medical Center—University Hospital Freiburg, 79106 Freiburg, Germany; 2 Faculty of Medicine, University of Freiburg, 79106 Freiburg, Germany; 3 Department of Obstetrics and Gynecology, St. Josefs-Hospital Wiesbaden, 65189 Wiesbaden, Germany; 4 Institute of Medical Biometry and Statistics, Medical Center – University of Freiburg, 79104 Freiburg, Germany; 5 Department of Gynaecology and Obstetrics, Hospital Krumbach, 86381 Krumbach, Germany; 6 Department of Obstetrics and Gynaecology, University Hospital of Basel, 4056 Basel, Switzerland; 7 Department of Gynaecology and Obstetrics, Diako Mannheim, 68135 Mannheim, Germany

**Keywords:** microRNAs, circulating microRNAs, urinary microRNAs, CDK inhibitor, palbociclib, breast cancer, disease biomarker, therapy response

## Abstract

Background: Breast cancer is the most common type of cancer worldwide. Cyclin-dependent kinase inhibition is one of the backbones of metastatic breast cancer therapy. However, there are a significant number of therapy failures. This study evaluates the biomarker potential of microRNAs for the prediction of a therapy response under cyclin-dependent kinase inhibition. Methods: This study comprises the analysis of intracellular and extracellular microRNA-expression-level alterations of 56 microRNAs under palbociclib mono as well as combination therapy with letrozole. Breast cancer cell lines BT-474, MCF-7 and HS-578T were analyzed using qPCR. Results: A palbociclib-induced microRNA signature could be detected intracellularly as well as extracellularly. Intracellular miR-10a, miR-15b, miR-21, miR-23a and miR-23c were constantly regulated in all three cell lines, whereas let-7b, let-7d, miR-15a, miR-17, miR-18a, miR-20a, miR-191 and miR301a_3p were regulated only in hormone-receptor-positive cells. Extracellular miR-100, miR-10b and miR-182 were constantly regulated across all cell lines, whereas miR-17 was regulated only in hormone-receptor-positive cells. Conclusions: Because they are secreted and significantly upregulated in the microenvironment of tumor cells, miRs-100, -10b and -182 are promising circulating biomarkers that can be used to predict or detect therapy responses under CDK inhibition. MiR-10a, miR-15b, miR-21, miR-23a and miR-23c are potential tissue-based biomarkers.

## 1. Introduction

Breast cancer (BC) is the most common type of cancer worldwide. With almost 700,000 deaths, it is also among the leading cancers in terms of mortality [1]. However, these data do not reflect its heterogeneity regarding histology, molecular biology and genomics, nor the adjunctive subtype-specific varying prognosis [2,3]. Histologically, lobular, ductal, mucinous, tubular, medullary and papillary breast carcinomas are classified [4,5,6,7]. Molecularly, BC is differentiated into luminal A, luminal B, HER2-enriched and triple-negative subtypes depending on positivity for estrogen receptors (ERs), progesterone receptors (PRs), the overexpression of human epidermal growth factor receptor 2 (HER2) and the tumor proliferation index level [8,9,10,11,12]. BC cells are further characterized according to the grade of differentiation (Grade 1–3) [13]. In BC therapy, multimodality is state-of-the-art, including surgery, radiotherapy, endocrine therapy, chemotherapy and several targeted therapies depending on various disease characteristics such as TNM status and molecular biology [2,14]. As a result of genomic cancer profiling, detailed subclassification of BC is possible depending on specific cell surface proteins, genes and other markers with prognostic and therapeutic relevance, enabling individualized cancer therapies [15].

One of the generally known hallmarks of cancer described by Hanahan and Weinberg is dysregulated cellular proliferation [16]. This has been shown in BC as well [17]. In particular, cyclin-dependent kinases (CDKs) are well studied in BC, resulting in the discovery of CDK4 and CDK6 as key drivers of aberrant cellular proliferation [17,18]. As a consequence, therapeutic targeting of the latter has led to the most fundamental change in the management of metastatic BC in the past decade, with striking improvements in progression-free survival (PFS). Palbociclib, ribociclib and abemaciclib are highly selective, orally bioavailable CDK4/6 inhibitors (CDKis) [17]. They demonstrate the ability to stop BC cells from progressing into the S phase of the cell cycle [17]. Initially, the PALOMA-1/TRIO-18 trial demonstrated a PFS prolongation of ten months (20.2 months vs. 10.2 months, HR 0.49, *p* = 0.0004) by adding palbociclib to letrozole compared to a letrozole monotherapy in patients with advanced ER-positive and HER2-negative BC [19]. This was validated by the PALOMA-2 study [20]. Additionally, the subsequent PALOMA-3 study also attested to the efficacy of palbociclib with fulvestrant in advanced ER-positive and HER2-negative BC with endocrine resistance [21]. Similar results were shown for ribociclib in the MONALEESA-2 trial [22] and for abemaciclib in the MONARCH-3 trial [23]. Clinical routine shows that the individual benefit of CDKi therapy is highly variable. Primary and secondary resistance limit their use for special groups of patients. The search for molecular biomarkers comprised elaborate analyses on the retinoblastoma protein (RB), cyclin D1, INK4 proteins and other proteins included in CyclinD1-CDK4/6-mediated pathways, as well as gene expression alterations, primarily CCNE1 [17,24]. Unfortunately, none of them showed promising potential. Further studies are needed to elucidate clinical or molecular biomarkers to predict CDK4/6i sensitivity, therapy responses or drug resistance.

MicroRNAs (miRs) are small non-coding single-stranded nucleotides. By modulating their targets, miRs are key players in tumorigenesis, disease progression and metastasis. They can act as both tumor suppressors or oncogenes [25,26]. Because they facilitate tumor progression and metastasis, miRs and circulating miRs in particular have emerged as BC biomarkers [25,27] and subtype classifiers [25,28]. Extensive studies in the past decade on their role in BC have elucidated their biomarker function for prognosis, diagnosis and therapy prediction in BC and metastatic BC. A new milestone is their potential as biomarkers for therapy responses and drug resistance, as well as their therapeutic impact on preventing metastasis development and altering drug resistance.

Because CDKis are new players in BC management, knowledge on the role of miRs is still limited. In order to understand the potential roles of miRs related to CDKis, it is important to describe their mode of action. In general, miRs interfere with the cyclin D1–CDK4/6–RB pathway by initiating the G1-S phase transition [17]. Interestingly, the cyclin D-CDK4/6 complex acts like a hub for many oncogenic pathways, e.g., the EGFR, HER2 and ER signaling pathway, as well as the PI3K–mTOR axis [29,30,31]. The role of miRs in this system has not been sufficiently investigated yet. MiRs-223, -3613-3p and miR-126, as well as the miR-106b cluster (miR-106b, miR-25, miR-93), exhibit interactions with CDKi-mediated pathways. In an in vitro and in vivo study, miR-223 and CDKi showed oppositional impacts on each other. On the one hand, CDKi therapy led to the upregulation of miR-223, whereas the downregulation of miR-223 promoted CDKi resistance in the same study. Citron et al. hypothesized that E2F1 might be responsible for this observation because it is a known repressor of miR-223 [32]. In a cell culture and tumor tissue study on TNBC, miR-3613-3p was directly linked to palbociclib sensitivity [33]. miR-126 showed CDKi-regulating properties in a study on BC cell lines [34]. Another study reported that the miR-106b cluster (miR-106b, miR-25, miR-93), as well as miRs-324-5p, -324-3p, -494, -99b and -671, is downregulated by CDK4/6 inhibition [35]. Additionally, there are several mainly cell-culture-based studies with significant evidence that there are interactions between miRs and CDK4/6 inhibition in other tumor entities, including atypical teratoid rhabdoid tumors, anaplastic large-cell lymphoma, prostate cancer, mantle cell lymphoma, lung cancer, renal cell carcinoma, melanoma, cutaneous squamous cell carcinoma, cervical cancer and gastric cancer. Specifically, these studies have reported interactions between CDKi and miRs-4270, the -17HG cluster, -497, -193b, -29, -3619-5p, 145, -1236, -365, -483_3p, -200a, -1, -34a and 143 [36,37,38,39,40,41,42,43,44,45].

**Figure 1:**
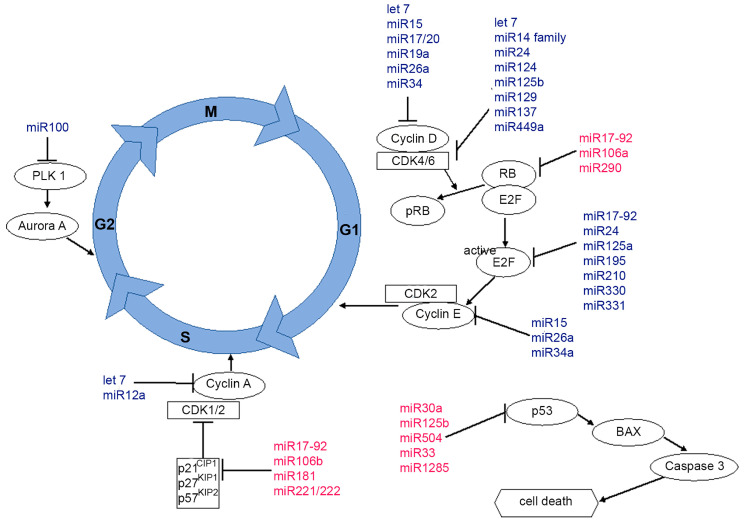
Overview of miRs involved in the cell cycle. miRs with proliferative effects are shown in red, and those with an antiproliferative effects are shown in blue.

Because there are only a few reports on the role of miRs in CDK4/6-directed therapy so far, there might be an opportunity to identify new biomarkers or therapeutic targets. miRs have been shown to play an important role in cell cycle regulation in general as well as in BC [46] via interaction with cyclins, CDKs, Rb, E2F and CDKi [46,47,48]. The miR-15a/16 cluster regulates many genes in the cell cycle, including cyclin D1, cyclin E1, cyclin D3 and CDK6 [49]. The miR-17/20 cluster is also involved in the transition from the G1 to the S phase, driven by the inhibition of E2F translation as well as by targeting the Rb protein and cyclin D1 [46,47,48]. In contrast, miRs-221 and -222 lead to tumor growth by inhibiting CDKis and by activating CDK2 [50]. Moreover, the let-7 family downregulates several cyclins, CDKs and their underlying genes, leading to cell cycle arrest [51,52]. The miR-34 family has also shown such properties via the p53 pathway [53]. In addition, earlier studies demonstrated that miR-137, miR-124a, the miR-192/215 cluster, the miR-194 cluster, the miR-17-92 cluster and miR-449 show the direct modulation of actors in cyclin/CDK-mediated cell cycle progression [54]. Regarding regulators of the aforementioned pathway, miR-106b-25, miR-221, miR-222, miR-24 and miR-181a target members of the Cip/Kip family and the INK4a/ARF family [54]. miRs-126, -21, -214, -216a, -217 and -192 and the miR-17-92 family are regulators of the upstream PI3K/AKT pathway [54]. However, these findings are not BC-specific. An overview of miRs involved in cell cycle regulation is provided in Figure 1.

The aim of the present study was to investigate the influence of CDK4/6 therapeutics on miR expression patterns in BC cells and their corresponding cell culture media. This could serve to develop biomarker signatures for therapy responses and might disclose specific miRs involved in the therapy mechanism underlying CDKis. Furthermore, the analysis of cell culture media provides insight into CDKi-mediated impacts on the tumor microenvironment, a key player in tumorigenesis and tumor progression.

## 2. Materials and Methods

### 2.1. Cell Culture Conditions and Treatments

The three established BC cell lines, BT-474, HS-578T and MCF7 (Cell Lines Service, Eppelheim, Germany, Table 1), were cultured in a humified incubator at 37 °C with a 5% CO_2_ atmosphere. The BT-474 cells (F12 medium with 5% calf serum, 1% Hepes and 1% Penicillin/Streptomycin), HS-578T cells (F12 medium, enhanced with 10% calf serum, 1% Hepes and 1% Penicillin/Streptomycin) and MCF7 cells (RPMI medium, containing 5% calf serum, 1% Hepes and 1% Penicillin/Streptomycin) were cultured in 25 mL cell culture flasks according to their specific known culturing conditions. Cell culture flasks with a growing surface of 25 cm^2^ (Greiner Bio-One, Frickenhausen, Germany) were for further experiments. In each experiment, 3.5 × 10^5^ cells were seeded and incubated for 24 h before treatment. Cells were checked for vitality under a microscope before treatment. Treated and untreated cells were cultured in parallel for direct comparison. Our experimental approach comprised a set of three different treatments plus two controls: the control (containing the cell specific culture medium only), control/DMSO, palbociclib, letrozole and palbociclib/letrozole. The dosage and timing of the treatments were carefully determined according to literature research and the performance of a cell viability MTT assay from prior testing with t-tests (3-(4,5-dimethylthiazol-2-yl)-2,5-diphenyltetrazolium bromide) and a tetrazolium reduction assay [55]. As shown by the MTT assay, palbociclib led to cell death in all three cell lines, indicating a response to the treatment. The minimal dosage that led to cell death in 50% of the cells after 24 h was 500 nM. Triplicates were generated. The letrozole concentration was calculated in the same way according to the findings of Desta et al. and was rounded to 300 nM [56]. These concentrations were used to treat the cell lines for further testing, in all treatments. After treatment, the samples were incubated for either 24 or 48 h.

### 2.2. RNA Isolation and Reverse Transcription

For intracellular RNA isolation, cells were lysed in 1 mL of RNA Extracol followed by the addition of 200 µL of chloroform and centrifugation at 13,000 rpm for 15 min at 4 °C for phase separation. Afterward, RNA isolation was performed by applying a EURx^®^ GeneMATRIX Universal RNA/miR purification kit (EURx Sp. Z o.o., Gdańsk, Poland) based on a method using total RNA mini spin columns. RNA was eluted with 50 µL of RNAse-free water and stored at −20 °C.

The isolation of extracellular RNA from miR-loaded exosomes or microvesicles followed a different protocol. First, the cellular growth medium was centrifuged at 4000 rpm for 10 min at 4 °C to remove cell debris and other residuals. Subsequently, the obtained supernatant was filtered using a 0.2 µm pore size filter. These filters were washed with 3 mL of DPBS and lysed with 500 µL of a lysis buffer containing 5 fM of two exogeneous synthetic “spike-in” control RNAs (Caenorhabditis elegans cel-miR39 and Arabidopsis thaliana ath-miR159). Then, 1200 µL of EtOH was added. The described mix was loaded onto a silica matrix micro spin column and centrifuged at 10,000 rpm. Three washing steps followed. The obtained RNA was eluted with 35 µL of RNAse-free water and stored at −20 °C. Subsequently, the obtained RNA concentration was determined spectrometrically (Nanophotometer^®^ N60). The next step comprised the reverse transcription (RT) of 500 ng of total RNA (miScript Reverse Transcription Kit, Qiagen GmbH, Hilden, Germany) to generate cDNA of miRs only. After reverse transcription, cDNA was diluted 1:10.

### 2.3. Quantitative PCR (qPCR)

Quantitative real-time polymerase chain reaction (qPCR) determined the miR expression levels in all aforementioned cell culture samples. Therefore, the Roche LightCycler 480 II (Roche applied Science, Mannheim, Germany) was used. Duplicates of each sample were examined. For qPCR, 1 µL of cDNA and 9 µL of an in-house qPCR mastermix (containing TRIS pH 8.1, dATP, dCTP, dGTP, dTTP, 5 mM magnesium, 15 mM potassium acetate, 10 mM ammonium sulphate, 1 µM SYBRGreen (Jena Bioscience, Jena, Germany), 0.01% BSA, 0.01% TRITON X100, HotStart Taq Polymerase (Jena Bioscience)) were used. A negative control (10 µL mastermix, no cDNA) and a minus-RT control (no RNA for reverse transcription, 1 µL unspecific cDNA, 9 µL mastermix) were added in order to evaluate if specific or unspecific products were amplified. Primers used for qPCR were designed using the miRNA qPCR primer design tool from Busk et.al. [57]. For primer sequences, see Supplemental Data S1.

To analyze miRNA expression, miR16, miR26b and U48 were used as endogenous controls. Delta Ct values were calculated using the comparative Ct method of Schmittgen and Livak [58]. Extracellular miRNA quantification was calculated according to Marabita et al. [59].

### 2.4. miR-Specimen

The analyzed miRs were as follows: let-7a-5p, let-7b-5p, let-7c-5p, let-7d-5p, let-7e-5p, let-7f-5p, miR-10a-5p, miR-10b-5p, miR-15a-5p, miR-15b-5p, miR-17-5p, miR-18a-5p, miR-19a-3p, miR-19b-3p, miR-20a-5p, miR-20b-5p, miR-21-5p, miR-23a-3p, miR-23c-3p, miR-25-5p, miR-26a-5p, miR-26b-5p, miR-29a-3p, miR-29c-3p, miR-30a-5p, miR-30b-5p, miR-30c-5p, miR-30e-5p, miR-92a-3p, miR-100-5p, miR-103-5p, miR-106b-5p, miR-107-3p, miR-122-5p, miR-125a-5p, miR-125b-5p, miR-126-3p, miR-128-3p, miR-148-3p, miR-181b-5p, miR-182-5p, miR-185-5p, miR-191-5p, miR-192-5p, miR-194-5p, miR-195-5p, miR-200a-3p, miR-200b-3p, miR-200c-3p, miR-210-3p, miR-221-3p, miR-222-3p, miR-223-3p, miR-301a-3p, miR-424-3p and miR-451a (primer sequences in Supplemental Data S1).

### 2.5. Analysis and Statistics

For the estimation and statistical testing of treatment effects, each compartment, treatment duration and miR was analyzed separately using two linear models. The first model was used for the analysis across all cell lines and comprised treatment effects as well as treatment-independent cell line effects to account for the different baseline levels in each cell line. The resulting treatment coefficients represent the joint treatment effects averaged over all cell lines. Therefore, we applied a multivariable linear regression model to the log-transformed expression values with the cell line (MCF7, BT-474 and HS-578T) and its two-way and three-way interactions with the treatment (control, control/DMSO, palbociclib, letrozole and palbociclib/letrozole) and compartment (intra-/extracellularly) as independent variables. All statistical methods involved ΔCt values normalized against the mean value of miR16, miR26b and U48. To calculate the influence of palbociclib, the ΔCt values of letrozole and both together for treated and untreated probes were compared statistically, which is represented in the relative expression (=2^−ΔCT^). For the interpretation of the multivariable analysis, all miRNA expression levels were compared to the intercept. The intercept represents cell line BT-474 under control conditions in the intracellular compartment. In the second model, cell-line-specific treatment effects were included in addition to estimated treatment effects within each cell line individually. For both analyses, the data were fitted on the log scale. *p*-values < 0.05 were considered significant.

## 3. Results

The present study analyzed the influence of the CDKi palbociclib on the expression levels of 56 miRs (see the Section 2) for three BC cell lines. The detection of intracellular and extracellular alterations aimed to identify potential biomarkers, indicating a therapy response for CDKis. In general, we were able to reliably detect the 56 aforementioned miRs in the intra- and extracellular compartment. The present study revealed subtype and cell-line-specific individual miR expression patterns in the intracellular and extracellular compartment regarding the 56 analyzed miRs. Regarding the different treatments, palbociclib, letrozole and the combination of them led to a measurable miR expression level alteration in each cell line and each miR. These alterations also showed cell-type-specific patterns. Five miRs showed significant uniform miR expression level alterations across all three analyzed cell lines, which indicates not only subtype-specific BC expression patterns but also BC-general alterations.

Two types of analyses were performed. In the first analysis, general effects were examined in all three BC cell lines. In the second analysis, we examined treatment effects for the individual cell lines. The results are summarized in the following two paragraphs.

### 3.1. Intracellular miR Expression Levels

**Figure 2:**
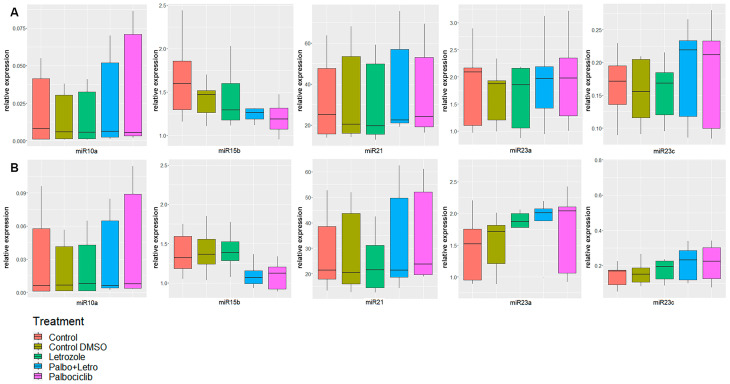
Significantly regulated microRNAs in the intracellular compartment, across all cell lines after a treatment duration of 24 h (**A**) and 48 h (**B**).

The analysis across all cell lines showed 39 significantly up- or downregulated miR expression levels irrespective of a co-treatment with letrozole (Table 2). A total of 21 of the aforementioned miRs showed significant expression level alterations under therapy with palbociclib plus letrozole but not under palbociclib alone (Table 2). Among these, ten miRs only became statistically significant after 48 h of treatment with both agents (let-7a-5p, let-7d-5p, let-7e-5p, miR-10b-5p, miR-20a-5p, miR-128-3p, miR-182-5p, miR-200b-3p, miR-200c-3p and miR-222-3p), suggesting time-dependent as well as treatment-dependent alteration causes. miR-106b-5p (CI: 0.55–0.87; *p*: 0.003) and miR-301a-3p (CI: 0.38–0.99; *p*: 0.046) only showed significant expression level alterations after 24 h of the palbocilcib plus letrozole therapy. Both were downregulated compared to the intercept. Eleven miRs (miR-25-5p, -29a-3p, -29c-3p, 30b-5p, -30c-5p, -30e-5p, -126-3p, -192-5p, -194-5p, -223-3p and -451a) were up- or downregulated under monotherapy with palbociclib after 24 h but not after the addition of letrozole. Only five miRs showed up- or downregulation under both therapy regimens: miR-10a-5p, miR-21-5p, miR-15b-5p, miR-23a-3p and miR-23c-3p. However, only miR-10a-5p showed constant upregulation under all four different treatments. Furthermore, each of the five miRs showed uniform upregulation (miR-10a-5p, miR-21-5p, miR-23a-3p and miR23c-3p) or uniform downregulation (miR-15b-5p), irrespective of the duration of the treatment and irrespective of the addition of letrozole (Table 3 and Figure 2 for confidence intervals (CI), *p*-values and boxplots). Letrozole alone did not lead to statistically significant alterations in any of the analyzed miRs.

The analysis with cell-line-specific coefficients revealed that miR regulation under palbociclib therapy appeared to be cell-line-specific. Here, the duration of the treatment was an important influencing factor as well. Table 4 provides a complete overview of all regulated miRs under palbociclib therapy. Obviously, like in untreated cells, the applied treatment leads to highly cell-line-specific miR expression patterns.

Interestingly, in BT-474 cells, in the palbociclib-mono-24 h treatment arm, there were 35 regulated miRs, whereas there were no significant changes after 48 h, and only miR-26b (upregulated, CI: 1.07–1.33; *p*: 0.002) was regulated in the combinatory treatment compared to the intercept. The MCF7 cells showed a contrary behavior: 16 miRs were regulated after 48 h of treatment with palbociclib plus letrozole. However, after 24 h, seven miRs were regulated; after 48 h and the palbociclib-mono treatment, three miRs were regulated; and after 24 h and the palbociclib-mono treatment, only miR-10a-5p (CI: 1.10–1.93; *p*: 0.010) was upregulated compared to the intercept. Hs-578T cells were constantly regulated across all treatment regimens.

In order to be able to draw clinical conclusions, it is necessary to analyze in more detail only those miRs that are regulated in the hormone-receptor-positive cell lines BT-474 and MCF7. Considering this, eight miRs were exclusively regulated in the two-hormone-receptor-positive cell lines BT474 and MCF7 compared to the intercept: let-7b, let-7d, miR-15a, miR-17, miR-18a, miR-20a, miR-191 and miR-301a_3p (see Table 4).

Table 4 shows all regulations cell line specifically in the multivariate analysis. The values are estimates representing fold change of the single miR compared to the intercept. P: palbociclib; P + L: Palbociclib plus letrozole

### 3.2. Extracellular miR Expression Levels

For the extracellular compartment, the analysis of miR expression alterations under palbociclib therapy across all cell lines shows that, by pooling all analyzed cell lines, there were no significant alterations under the palbociclib monotherapy for 24 h. After the palbociclib monotherapy for 48 h, only miR-100 showed significant upregulation (CI: 1.05–1.56; *p*: 0.014). Treatment with palbociclib plus letrozole led to significant downregulation of miR-10b (CI: 0.63–1.01; *p*: 0.058) and significant upregulation of miR-182 (CI: 1.24–2.32; *p*: 0.002). Letrozole alone led to the downregulation of miR-128_3p (CI: 0.68–0.96; *p*: 0.015) and miR-210_3p (CI: 0.52–0.95; *p*: 0.021) and the upregulation of let-7f (CI: 1.07–1.56; *p*: 0.008).

**Figure 3:**
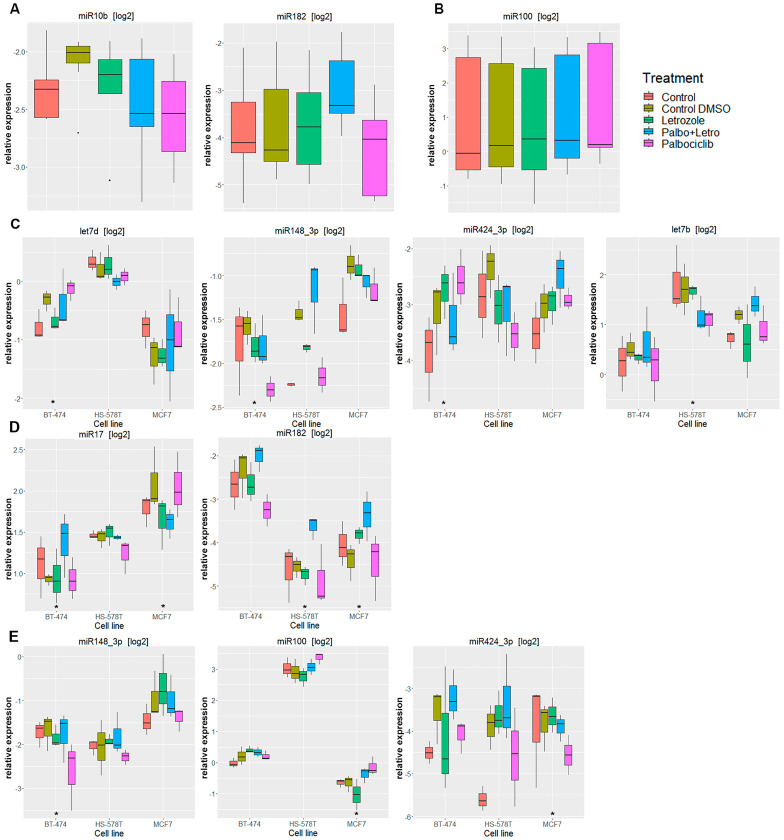
Significantly regulated microRNAs in the extracellular compartment for all cell lines. (**A**) After 24 h of treatment; (**B**) After 48 h of treatment. Selection of significantly regulated microRNAs in the extracellular compartment for individual cell lines. The cell line with significant regulation was labeled with a *: (**C**) After 24 h of treatment with Palbociclib; (**D**) After 24 h of treatment with palbociclib + letrozole; (**E**) After 48 h of treatment with palbociclib.

The analysis with cell-line-specific coefficients for miR expression alterations under palbociclib therapy in the extracellular compartment revealed results that were comparable to those from the intracellular compartment: cell-line-specific miR expression alterations depended on the duration and combination of the treatment. Table 5 provides an overview of all significantly altered miRs in the extracellular compartment. The three miRs miR-17, miR-148_3p and miR-424_3p showed promising expression patterns. Compared to the intercept, miR-17 was downregulated, and miR-424_3p was regulated bidirectionally in hormone-receptor-positive cell lines. miR-148_3p was altered in the extracellular compartment of BT-474 only. Figure 3 shows box plots of the relevant miRs that were found to be significantly altered across all cell lines and for the cell-line-specific analysis extracellularly.

### 3.3. Duration of Cell Culture Treatment

Regarding the duration of the treatment with palbociclib or palbociclib plus letrozole, there was no definite duration at which cells responded more or less clearly to the treatment, irrespective of the analyzed compartment. Interestingly, this seems to be cell-line- as well as miR-specific. For the analysis with cell-line-specific effects, it is possible to correlate the duration of the treatment (24 h and 48 h) with each cell line exclusively. Descriptively, the BT474 cell line seemed to be more regulated only when treated for 24 h, whereas the MCF7 cell line seemed to be more regulated when treated for 48 h. The triple-negative BC cell line Hs-578T showed equal results in the 24 h and 48 h treatment approaches. Furthermore, the duration of treatment led to expression level alterations in different miRs in the same cell lines (see Table 4). In the univariate analysis in the intracellular compartment, miR-20b (palbociclib mono: CI: 0.58–0.99; *p*: 0.045; palbociclib plus letrozole: CI: 0.56–0.96; *p*: 0.027) and miR-100 (palbociclib mono: CI: 1.02–1.49; *p*: 0.030; palbociclib plus letrozole: CI: 1.00–1.47; *p*: 0.046) exhibited a regulatory effect under palbociclib monotherapy and under the palbociclib plus letrozole combination, both exclusively after 48 h.

Table 6 shows a summary of significantly regulated miRs under palbociclib therapy and their potential role reported in previous studies.

## 4. Discussion

By reviewing the existing body of literature, it can be seen that many miRs described in context with CDKis or the cell cycle in general did not show promising results in the preliminary screening procedures of our study, or their detection was simply not steady enough. However, by comparing existing studies and our results, it can be seen that 8 of the 56 analyzed miRs have been described in context with CDKis (miR-17, miR-25, miR-29, miR-92, miR-106b, miR-126, miR-200a and miR-223), and 17 have been described in context with the cell cycle in general (miR-15a, miR-16, miR-17, miR-20, miR-92, miR-21, miR-126, miR-192, miR-194, miR-221 and miR-222).

As shown in a variety of previous studies, miR expression patterns are subtype-specific [28]. Our study confirms this hypothesis. Regarding the miR expression patterns of the three analyzed cell lines in the untreated condition, there are highly individual miR signatures for each of them. Moreover, the presented study also confirms the feasibility of reliably detecting miRs in the extracellular compartment of cell cultures. This has also been shown in a previous study [27]. However, the aim of the present study was to evaluate the therapeutic effect of CDKi on BC cells (intracellular expression level alterations) and how this effect could be detected in the extracellular compartment. The evaluation of extracellular expression level alterations after palbociclib therapy is therefore expected to help identify a potential non-invasive miR biomarker for CDKi therapy responses. Intracellular expression level alterations rather depict a general potential and might serve to establish therapy predictions. In general, the results displayed above definitely depict the therapeutic effect of palbociclib in the form of miR expression alterations, intra- and extracellularly. These alterations are cell-line- and therefore subtype-specific, as well. Cell culture studies on secreted miRs with biomarker purposes regarding a CDK-directed therapy are not yet available. Furthermore, a large-scale analysis on miR expression patterns indicating treatment with a CDKi are scarce, as well. As a result, knowledge on potential miR biomarkers with such a purpose is unsatisfactory. By reviewing the current body of literature, it can be seen that approximately 27 miRs or miR clusters have been directly associated with CDK-4/6 therapy in BC [32,33,34,35] or cancer in general [36,37,38,39,40,41,42,43,44,45]. Of the 27 aforementioned miRs, only 8 (miR-17, -25, -29, -92a, -106b, -126, -200a, -223) were reliably and reproducibly detectable in the screening phase of our study. The large discrepancies between the existing studies themselves and the presented study mostly arise from methodological differences. Cell-culture-based miR analyses comprise a five-step procedure until miR expression levels can be quantified: cell culturing, treatment, RNA isolation, reverse transcription and miR detection, mostly via qPCR. Because standardized methods are missing, and due to the large number of available preparation kits for each step, there is huge interstudy variability of the results.

The analysis across all cell lines revealed that miR-10a-5p, miR-15b-5p, miR-21-5p, miR-23a-3p and miR-23c-3p were intracellularly significantly regulated across all cell lines under at least one of the four applied therapeutic algorithms. miR-10a-5p showed constant upregulation under the palbociclib and palbociclib combination therapy with letrozole after 24 h and 48 h. In different studies, miR-10a showed a tumor-suppressive function targeting the PI3K/Akt/mTOR signaling pathway [59,60]. The PI3K/AKT/mTOR pathway is a key regulator of the cell cycle [61]. Downstream, it inhibits the cell cycle, targeting its most crucial actors: Rb and Cyclin D1, among others [61]. As such, it has a tumor-suppressive function. Moreover, Khan et al. analyzed the miR-10a content in the tumor tissue of 103 BC patients. Compared to healthy control breast tissues, the miR-10a quantity was significantly reduced, suggesting its tumor-suppressive role [81]. In a BC-tissue-based study by Hoppe et al., increased miR-10a expression was correlated with a longer relapse-free period after tamoxifen therapy [82]. Finally, in a liquid-biopsy-based study on patient plasma, miR-10a was downregulated in triple-negative as well as hormone-receptor-positive BC patients [83]. These findings are in line with the detected upregulation under CDKi therapy in our study. In hormone-receptor-positive and triple-negative BC cells, miR-10a was constantly upregulated across all treatment algorithms. The increased intracellular expression of miR-10a after palbociclib therapy suggests that miR-10a upregulation is a result of the inhibition of CDK4/6 and therefore cell cycle arrest. The downregulation of cell proliferation leads to the upregulation of miR-10a expression, which underlines its tumor-suppressive feature. On the contrary, there are also in vitro and in vivo studies demonstrating that elevated miR-10a levels correlate with disease recurrence and chemoresistance [84,85,86], indicating an oncogenic role. These oppositional results mirror the second unsolved problem besides the methodological difficulties, which hinders the routine use of miRs as cancer biomarkers, namely the diversity of the functions of one single miR. This is based on the fact that one miR can regulate several complementary mRNAs and that it can also function epigenetically. Furthermore, these various functions of single miRs are not completely understood, leading to the third difficulty in interpreting the results of different studies. Under cell culture conditions as well as in vivo, a vast number of cellular mechanisms and pathways are simultaneously ongoing. All these mechanisms hypothetically create a specific miR pattern, and the entirety of simultaneous cellular mechanisms leads to an miR signature that differs from time to time. It represents a dynamic process [87].

Intracellularly, four more miRs were altered across all cell lines under the palbociclib and palbociclib plus letrozole treatments: miR-15b, miR-21, miR-23a and miR-23c. In BC, miR-15b has shown mostly oncogenic features [62,63,88]. Generally, in vitro and in vivo experiments have revealed upregulated expression of miR-15b in BC [62,88]. Among its potential reported targets are HPSE2 (heparanase-2) [88], PAQR3 (Progestin And AdipoQ Receptor Family Member 3) [62] and MTSS1 (metastasis suppressor protein 1) [89]. All of them are tumor suppressors. Interestingly, miR-15b also regulates the WEE1 (Wee1-like protein kinase) gene, a key regulator of the cell cycle [64]. In the present study, miR-15b was downregulated after palbociclib therapy as well, which also indicates its oncogenic role. miR-21 is widely known as oncogenic [65]. As such, there is plenty of evidence that it is upregulated in various cancers, including BC [65]. One of its targets is the PI3K/AKT-pathway, which is directly connected to the cell cycle [54]. The overexpression of miR-21 leads to enhanced cancer cell proliferation via this pathway [90]. Upregulated miR-21-levels in our study might be explained by two main factors. First, other cancer-related mechanisms are still ongoing, and second, the upregulation of miR-21 is a result of the BC cells counter steering the palbociclib effect. Finally, in the univariate analysis of the study, miR-23a and miR-23c were upregulated under the palbociclib treatment. Regarding miR-23a, there are numerous reports on the expression patterns in different cancer entities. Whereas it is downregulated in endometrial and prostate cancer, it is upregulated in BC [66,91]. Although extensively studied, there are only a few studies on its potential targets in BC. Interestingly, it was demonstrated that the suppression of miR-23a leads to the inhibition of BC metastasis and invasion [92]. miR-23c is studied less frequently in BC. However, there is evidence of its tumor-suppressive function [67]. These findings are in line with the detected upregulation after the palbociclib treatment.

The major aim of this study, however, was to identify potential liquid-biopsy-based predictive biomarkers for palbociclib therapy. Therefore, the analysis of the extracellular compartment of the different cell lines became necessary. We found that miR-100, miR-10b and miR-182 were significantly upregulated under the palbociclib therapy across all analyzed cell lines. miR-100 is one well-studied miR in BC. In general, miR-100 has shown tumor-suppressive features in the greater part of the existing literature [69,93,94] by suppressing tumorigenesis and invasion. Furthermore, miR-100 was found to sensitize BC cells to paclitaxel and hormonal treatment [95,96]. As a disease biomarker, miR-100 has proven potential in a tissue-based study, where it was downregulated [97]. Furthermore, it has proven potential in predicting endocrine responsiveness [98]. Interestingly, it might also serve as a circulating biomarker for therapy responses under tyrosine kinase inhibitors [99]. miR-10b is also known as a tumor suppressor targeting the cell cycle via several target genes [100]. Regarding its biomarker role, it has proven potential, like miR-100, in the same tissue-based study, where it was downregulated as well [97]. As a circulating biomarker in body liquids, miR-10b also exhibited an association with drug responses and was upregulated in the plasma of BC patients [101]. miR-182 was found to be upregulated in the aforementioned and other studies and is generally known as an oncogene [97,102]. As a circulating biomarker, it has been studied in different approaches. In a plasma-based study, it did not show diagnostic value [103]. However, it was upregulated in another study on the blood of BC patients [104]. Interestingly, it has been shown to regulate drug resistance in BC [105].

These findings qualify each of them as well as their combination as potential biomakers in liquid biopsies for therapy responses or predictive biomarkers of CDK-4/6 therapy in BC. However, because in clinical routine only hormone-receptor-positive BC patients receive CDKis, the results of the cell-line-specific analysis might reveal promising miR-biomarkers with such a purpose. miR-17 was downregulated exclusively in hormone-receptor-positive cells. miR-17 is described as an oncogene and was downregulated in the sera of BC patients [106]. In a prior analysis by our group, it also showed promising potential as a circulating biomarker for a therapy response in triple-negative BC [107], where it was also downregulated.

miR-92 was exclusively regulated extracellularly in the HR-positive and HER2-negative cell line MCF7 under the combination therapy with palbociclib and letrozole after 24 h of treatment. miR-92 is part of the miR17-92 cluster, which has an oncogenic role [108]. Upregulation of miR-92 is also associated with tumor progression in breast cancer [109]. In breast cancer, miR-92 negatively regulates the expression of estrogen receptor beta 1 [79]. We were able to show the downregulation of miR-92 extracellularly in the HR-positive cell line MCF-7 under treatment with palbociclib and letrozole after 24 h. This could indicate an early therapy response.

As in plenty of other studies on miRs in BC in general, as well as under BC therapy, this study found cell-line-specific miR patterns as well as subtype-specific deregulation patterns. By comparing the deregulated miRs in the hormone-receptor-positive cell lines BT-474 and MCF to the ones in the triple-negative BC cell line Hs-478T, it can be seen that miR let-7b, let-7d, miR-15a, miR-17, miR-18a, miR-20a, miR-191 and miR-301a_3p are differentially expressed. Let-7c, miR-25, miR-26b, miR-106b, miR-125a, miR-125b and miR-181b account for subtype specificity. Prior studies justify this subtype specificity with histologic, genetic and molecular features [80,110,111].

Finally, our study also reveals that the duration of palbociclib treatment and its co-therapy with letrozole led to specific miR expression level alterations themselves. To the best of our knowledge, there is no study that evaluates the length of treatment under palbociclib and its different results. By summing our experiments up, it becomes clear that the miR expression patterns in the four treatment arms are highly individual. A study conducted by Bozkurt et al., however, supports our findings, stating that miR expression alterations are dynamic and time-dependent [87]. Furthermore, the study is also able to conclude that let-7f, miR-128_3p and miR-210_3p are promising potential biomarkers for therapy responses under letrozole therapy, because these were significantly upregulated across the three BC cell lines in the extracellular compartment. Let-7f was shown to be upregulated after an aromatase inhibitor therapy in another study, as well [68,112,113].

The limitations of this study are the limited number of analyzed cell lines. In order to draw conclusions on subtype-specific alterations under palbociclib therapy, a larger number of cell lines of different molecular backgrounds should be analyzed. An additional analysis of miRNAs in palbociclib-resistant cell models also seems to be interesting. In order to correlate the presented miRNAs and biomarker functions in liquid biopsies, a patient sample analysis would be necessary to prove their potential as biomarkers in vivo. Furthermore, the drugs ribociclib and abemaciclib should also be analyzed.

## 5. Conclusions

Palbociclib treatment leads to a detectable miR expression level alteration in BC cell lines as well as in their corresponding microenvironment. The treatment can be footprinted as a specific miR signature. Constant intracellular alterations over all analyzed cell lines qualify miR-10a, miR-15b, miR-21, miR-23a and miR-23c as potential biomarkers for therapy responses or predictions. In the extracellular compartment, miR-100, miR-10b and miR-182 were constantly altered under palbociclib therapy. As a consequence, this panel qualifies as a very promising biomarker tool for liquid biopsies to indicate therapy responses. Furthermore, these secreted miRs indicate a potential role in cell-to-cell communication and therefore in tumor-microenvironment-mediated tumor progression, making them promising targets for novel BC therapeutics. Similarly, the intracellular expression patterns of let-7b, let-7d, miR-15a, miR-17, miR-18a, miR-20a, miR-191 and miR-301a-3p and the extracellular downregulation of miR-17 in hormone-receptor-positive cells only potentially represent such biomarkers, because, clinically, only hormone-receptor-positive patients undergo therapy with CDKi. Furthermore, let-7f, miR-128_3p and miR-210_3p could serve as circulating biomarkers indicating an aromatase inhibitor therapy.

Prospectively, clinical studies on liquid biopsies should be undertaken in order to verify these cell culture findings in vivo with the aim of developing a biomarker tool for CDKi therapy predictions and responses. The greater goal should be to minimize the number of therapy failures and therefore improve BC survival and BC-associated quality of life.

## Figures and Tables

**Table 1:** Analyzed BC cell lines and their characteristics.

	BT-474	MCF7	Hs-578T
Histologic subtype	ductal	ductal	ductal
HER2 status	+	-	-
ER status	+	+	-
PR status	+	+	-
Cell origin	Breast tumor	Pleural effusion	Breast tumor

**Table 2:** Significantly regulated miRs across all cell lines (intracellular).

	24 h	48 h
Palbociclib	**miR-10a**, **miR-15b**, miR-25, miR-29a, miR-29c, miR-30b, miR-30c, miR-30e, miR-126, miR-192, miR-223, miR-194, miR-451a	**miR-10a**, **miR-15b**, miR-20b, **miR-21**, **miR-23a**, **miR-23c**, miR-25, **miR-100**, miR-126
Letrozole	-	-
Palbociclib + Letrozole	let-7c, **miR-10a**, miR-17, miR-18a, **miR-21**, **miR-23a**, **miR-23c**, miR-26a, miR-26b, miR-30a, miR-106b, miR-125a, miR-125b, miR-181b, miR-301a,	let7a, let7c, let7d, let7e, **miR-10a**, miR-10b, **miR-15b**, miR-17, miR-18a, miR-20a, miR-20b, **miR-21**, **miR-23a**, **miR-23c**, miR-26a, miR-26b, miR-30a, miR-100, miR-125a, miR-125b, miR-128, miR-181b, miR-182, miR-200b, miR-200c, miR-222

Overview of all significantly regulated miRs after treatment with palbociclib (top), letrozole (middle) and their combination (bottom). miRs regulated in both palbociclib and combinatory treatment appear in bold. All shown miRs showed significant alterations (*p* < 0.05) in the univariate analysis.

**Table 3:** Intracellular—regulation manner of the five constantly deregulated miRs: miRs-10a-5p, miR-15b-5p, miR-21-5p, miR-23a-3p and miR-23c-3p.

	24 h		48 h	
	P	P + L	P	P + L
miR-10a-5p	1.46 *p*-value: 0.01 CI: 1.10–1.93	1.57 *p*-value: 0.00 CI: 1.19–2.08	1.67 *p*-value: 0.00 CI: 1.27–2.19	1.52 *p*-value: 0.00 CI: 1.15–1.99
miR-15b-5p	0.75 *p*-value: 0.00 CI: 0.66–0.87	Not significant	0.82 *p*-value: 0.01 CI: 0.70–0.96	0.86 *p*-value: 0.05 CI: 0.72–1.01
miR-21-5p	Not significant	1.15 *p*-value: 0.01 CI: 1.03–1.28	1.22 *p*-value: 0.00 CI: 1.09–1.37	1.14 *p*-value: 0.03 CI: 1.01–1.28
miR-23a-3p	Not significant	1.14 *p*-value: 0.04 CI: 1.00–1.29	1.19 *p*-value: 0.04 CI: 1.00–1.42	1.21 *p*-value: 0.02 CI: 1.02–1.44
miR-23c-3p	Not significant	1.18 *p*-value: 0.03 CI: 1.01–1.37	1.49 *p*-value: 0.00 CI: 1.13–1.96	1.40 *p*-value: 0.01 CI: 1.06–1.84

Constantly regulated miRs under both therapy regimens (P: palbociclib; P + L: palbociclib plus letrozole) after a treatment duration of 24 h and 48 h, irrespective of the cell line. The top row shows the estimate representing the x-fold relative expression of the single miRs compared to the intercept; CI: confidence interval.

**Table 4:** miRs with significant cell-line-specific treatment effects for the analysis of intercellular expression levels.

miR	BT-474				MCF7				HS-578T			
Treatment Algorithm	P 24 h	P + L 24 h	P 48 h	P + L 48 h	P 24 h	P + L 24 h	P 48 h	P + L 48 h	P 24 h	P + L 24 h	P 48 h	P + L 48 h
let-7a-5p	−1.63											
let-7b-5p	−1.63							1.60				
let-7c-5p	−1.69							1.52		1.47		
let-7d-5p	−1.63							1.37				
let-7e-5p	−1.59											
let-7f-5p												
miR-10a-5p					2.41	2.15	2.83	2.13	1.67	1.71	1.45	1.47
miR-10b-5p	−1.75											
miR-15a-5p	1.41					−1.40						
miR-15b-5p	−1.63								−1.30		−1.37	
miR-17-5p	1.39							−1.69				
miR-18a-5p	1.83					−1.47		−3.03				
miR-19a-3p												
miR-19b-3p												
miR-20a-5p	1.53							−1.89				
miR-20b-5p								−1.69				
miR-21-5p							1.28			1.29	1.24	
miR-23a-3p									1.29	1.30		
miR-23c-3p								1.68	1.39	1.58	1.88	
miR-25-5p	−2.08						−1.35		−1.47		−1.45	−1.47
miR-26a-5p										1.20		
miR-26b-5p	−1.19	1.16				1.22			1.33	1.19		1.30
miR-29a-3p	1.44											
miR-29c-3p	1.76											
miR-30a-5p										1.44		1.40
miR-30b-5p	1.51											
miR-30c-5p										1.30		
miR-30e-5	2.22											
miR-92a-3p	−1.63											
miR-100-5p										1.53	1.37	1.41
miR-103-5p												
miR-106b-5p	1.43					−1.85			−1.52			
miR-107-3p												
miR-122-5p	−1.92											
miR-125a-5p	−1.75							1.69		1.75		
miR-125b-5p	−1.61							1.55		1.69		1.71
miR-126-3p	1.69								1.63	1.61	2.10	1.63
miR-128-3p	−1.49									1.34		
miR-148-3p	−1.35											
miR-181b-5p	−1.44							1.58		1.31		
miR-182-5p	−1.52								1.42	1.42		
miR-185-5p	−1.56											1.26
miR-191-5p	−1.69							1.49				
miR-192-5p									1.43	1.54		1.42
miR-194-5p	1.34								1.42	1.39	1.52	1.48
miR-195-5p												
miR-200a-3p						−2.04						
miR-200b-3p								1.57			1.48	
miR-200c-3p	−1.75											
miR-210-3p												
miR-221-3p	−1.47											
miR-222-3p												
miR-223-3p	−5.26											
miR-301a-3p	2.41					−2.5						
miR-424-3p												
miR-451a	1.66											

**Table 5:** Overview of significantly regulated miRs after analysis for the extracellular compartment.

	24 h		48 h	
	P	P + L	P	P + L
All cell lines	-	miR-10b miR-182	miR-100	-
BT-474	let-7d miR-148 miR-424	miR-17	miR-148	-
MCF7	miR-26a miR-26b	miR-17 miR-25 miR-92a miR-182	miR-100 miR-125a miR-424	-
HS-578T	let-7b miR-200a	miR-182 miR-200c	miR-200c miR-301a	miR-192

Overview of all significantly regulated miRs after treatment with palbociclib (P) and palbociclib plus letrozole (P + L). All shown miRs showed significant alterations (*p* < 0.05) in the univariate and multivariable analyses.

**Table 6:** Reported roles of significantly regulated, investigated microRNAs.

microRNA	Function	Reference
Intracellular		
miR-10a	Tumor suppressor by inhibiting PI3K/Akt/mTOR signaling pathway	[60,61]
miR-15b	Oncogenic by inhibiting tumor suppressors	[62,63,64,65]
miR-21	Oncogenic by promoting cell proliferation via the PTEN/PI3K/Akt pathway	[66]
miR-23a	Oncogenic in breast cancer, where it promotes tumor invasion and metastasis Tumor suppressor in pancreatic cancer, where it leads to apoptosis via inhibiting the PLK-1 expression in vitro	[67,68]
miR-23c	Tumor suppressor, inhibits cell proliferation by targeting the erb2 interacting protein in hepatocellular carcinoma	[69]
let-7b	Tumor suppressor by inhibiting cell proliferation	[70]
let-7d	Tumor suppressor by targeting Jab 1 in breast cancer	[71]
miR-15a	Tumor suppressor, associated with mitochondrial-mediated apoptosis via downregulation of the oncogene BMI 1	[72]
miR-17	Oncogenic by inducing cell proliferation	[73]
miR-18a	Tumor suppressor, inhibits cell proliferation in breast cancer	[74]
miR-20a	Oncogenic by negative regulation of PTEN	[75]
miR-191	Oncogenic, promotes proliferation of breast cancer cells via downregulation of DICER 1	[76]
miR-301a	Oncogenic, inhibits ESR1 expression in ER-positive breast cancer	[77]
Extracellular		
miR-17	Oncogenic, increased levels are associated with cell proliferation and poor prognosis in breast cancer	[78]
miR-92	Oncogenic, leads to breast cancer progression and inhibits expression of estrogen receptor β1 in HR-positive breast cancer	[74,79,80]

## Data Availability

The data presented in this study are available on request from the corresponding author. The data are not publicly available due to privacy.

## Supplementary Materials

The following supporting information can be downloaded at https://www.mdpi.com/article/10.3390/biomedicines11102705/s1: Supplemental Data S1: Primer sequences.

## Abbreviations

BC: breast cancer; CDK: cyclin-dependant kinase; CDKi: cyclin-dependant kinase inhibitor/inhibitors; CI: confidence interval; miR: microRNA; PFS: progression-free survival; qPCR: quantitative real-time polymerase chain reaction; RT: reverse transcription.
