# Supplementary material for: In Vitro microRNA Expression Profile Alterations under CDK4/6 Therapy in Breast Cancer
Source: Biomedicines. 2023 Oct 5;11(10):2705. doi: 10.3390/biomedicines11102705 (PMC10604872; doi:10.3390/biomedicines11102705)
Supplement: Supplementary file 1 [file biomedicines-11-02705-s001.zip › biomedicines-2551189-supplementary.pdf]

Supplemental data 1: Primer sequences

**cel-miR-39_3p**	**S:** 5'- GTC ACC GGG TGT AAA TCA G - 3'
	**AS:** 5'- GGT CCA GTT TTT TTT TTT TTT TCA AG - 3'
**ath-miR-159a**	**S:** 5'- GCG CAG TTT GGA TTG AAG - 3'
	**AS:** 5'- AGG TCC AGT TTT TTT TTT TTT TTA GAG - 3'
**U48**	**S:** 5'- TCA CCG CAG CGC TCT - 3'
	**AS:** 5'- TCC AGT TTT TTT TTT TTT TTG GTC A - 3'
**let7a**	**S:** 5' - GCA GTG AGG TAG TAG GTT G – 3'
	**AS:** 5' - GGT CCA GTT TTT TTT TTT TTT TAA CTA TAC – 3'
**let7b**	**S:** 5' - CAG TGA GGT AGT AGG TTG TGT - 3'
	**AS:** 5' - GGT CCA GTT TTT TTT TTT TTT TAA CCA - 3'
**let7c**	**S:** 5' - GCA GTG AGG TAG TAG GTT GT - 3'
	**AS:** 5' - GGT CCA GTT TTT TTT TTT TTT TAA CCA – 3'
**let7d**	**S:** 5' - CGC AGA GAG GTA GTA GGT TG - 3'
	**AS:** 5' - GGT CCA GTT TTT TTT TTT TTT TAA CTA TG - 3'
**let7e**	**S:** 5' - GCA GTG AGG TAG GAG GTT G - 3'
	**AS:** 5' - GGT CCA GTT TTT TTT TTT TTT TAA CTA TAC - 3'
**let7f**	**S:** 5' - CGC AGT GAG GTA GTA GAT TG - 3'
	**AS:** 5' - CAG GTC CAG TTT TTT TTT TTT TTT AAC - 3'
**miR10a**	**S:** 5' - CAG TAC CCT GTA GAT CCG AAT - 3'
	**AS:** 5' - GGT CCA GTT TTT TTT TTT TTT TCA C - 3'
**miR10b**	**S:** 5' - CAG TAC CCT GTA GAA CCG A - 3'
	**AS:** 5' - GGT CCA GTT TTT TTT TTT TTT TCA C - 3'
**miR15a**	**S:** 5' - CAG TAG CAG CAC ATA ATG GTT - 3'
	**AS:** 5' - GGT CCA GTT TTT TTT TTT TTT TCA CA - 3'
**miR15b**	**S:** 5' - GTA GCA GCA CAT CAT GGT T - 3'
	**AS:** 5' - CAG GTC CAG TTT TTT TTT TTT TTT GTA - 3'
**miR16**	**S:** 5'- CGC AGT AGC AGC ACG TA - 3'
	**AS:** 5'- CAG TTT TTT TTT TTT TTT CGC CAA - 3'
**miR17**	**S:** 5' - GCA AAG TGC TTA CAG TGC AG - 3'
	**AS:** 5' - GGT CCA GTT TTT TTT TTT TTT TCT AC - 3'
**miR18a**	**S:** 5' - GTA AGG TGC ATC TAG TGC AG - 3'
	**AS:** 5' - GGT CCA GTT TTT TTT TTT TTT TCT ATC - 3'
**miR19a_3p**	**S:** 5' - GCA GTG TGC AAA TCT ATG CAA - 3'
	**AS:** 5' - GGT CCA GTT TTT TTT TTT TTT TCA GT - 3'
**miR19b_3p**	**S:** 5'- AGT GTGCAA ATC CAT GCA - 3'
	**AS:** 5' - GGT CCA GTT TTT TTT TTT TTT TCA GT - 3'
**miR20a**	**S:** 5' - GCA GTA AAG TGC TTA TAG TGC AG - 3'
	**AS:** 5' - GGT CCA GTT TTT TTT TTT TTT TCT AC - 3'
**miR20b**	**S:** 5' - GCA AAG TGC TCA TAG TGC AG - 3'
	**AS:** 5' - GGT CCA GTT TTT TTT TTT TTT TCT AC - 3'
**miR21**	**S:** 5' - GCA GTA GCT TAT CAG ACT GAT G - 3'
	**AS:** 5' - GGT CCA GTT TTT TTT TTT TTT TCA AC - 3'
**miR23a_3p**	**S:** 5' - AGA TCA CAT TGC CAG GGA T - 3'
	**AS:** 5' - GGT CCA GTT TTT TTT TTT TTT TGG AA - 3'
**miR23c**	**S:** 5' - CAG ATC ACA TTG CCA GTG ATT - 3'
	**AS:** 5' - GTC CAG TTT TTT TTT TTT TTT GGG T - 3'
**miR25**	**S:** 5' - CAT TGC ACT TGT CTC GGT - 3'
	**AS:** 5' - GGT CCA GTT TTT TTT TTT TTT TCA G - 3'
**miR26a**	**S:** 5' - GCA GTT CAA GTA ATC CAG GAT AG - 3'

	**AS:** 5′ - GGT CCA GTT TTT TTT TTT TTT TAG C - 3′
**miR26b**	**S:** 5′ - CGC AGT TCA AGT AAT TCA GGA T - 3′
	**AS:** 5′ - GGT CCA GTT TTT TTT TTT TTT TAC CT - 3′
**miR29a**	**S:** 5′ - GCA GTA GCA CCA TCT GAA ATC - 3′
	**AS:** 5′ - CAG GTC CAG TTT TTT TTT TTT TTT AAC - 3′
**miR29c_3p**	**S:** 5′ - CAG TAG CAC CAT TTG AAA TCG - 3′
	**AS:** 5′ - GGT CCA GTT TTT TTT TTT TTT TAA CC - 3′
**miR30a**	**S:** 5′ - GCA GTG TAA ACA TCC TCG AC - 3′
	**AS:** 5′ - TCC AGT TTT TTT TTT TTT TTC TTC CA - 3′
**miR30b**	**S:** 5′ - GCA GTG TAA ACA TCC TAC ACT CA - 3′
	**AS:** 5′ - GGT CCA GTT TTT TTT TTT TTT TAG C - 3′
**miR30c**	**S:** 5′ - GCA GTG TAA ACA TCC TAC ACT CT - 3′
	**AS:** 5′ - GGT CCA GTT TTT TTT TTT TTT TCT GA - 3′
**miR30e**	**S:** 5′ - CGC AGT GTA AAC ATC CTT GAC - 3′
	**AS:** 5′ - TCC AGT TTT TTT TTT TTT TTC TTC CA - 3′
**miR92a**	**S:** 5′- CGC AGT ATT GCA CTT GTC - 3′
	**AS:** 5′- GGT CCA GTT TTT TTT TTT TTT TAC AG - 3′
**miR100**	**S:** 5′- CAG AAC CCG TAG ATC CGA - 3′
	**AS:** 5′- GTC CAG TTT TTT TTT TTT TTT CAC AAG - 3′
**miR103**	**S:** 5′ - GCA GAG CAG CAT TGT ACA G - 3′
	**AS:** 5′ - GGT CCA GTT TTT TTT TTT TTT TCA TAG - 3′
**miR106b**	**S:** 5′ - GCA GTA AAG TGC TGA CAG TG - 3′
	**AS:** 5′ - GGT CCA GTT TTT TTT TTT TTT TAT CTG - 3′
**miR107**	**S:** 5′ - GCA GAG CAG CAT TGT ACA G - 3′
	**AS:** 5′ - GGT CCA GTT TTT TTT TTT TTT TGA TAG - 3′
**miR122**	**S:** 5′- GCA GTG GAG TGT GAC ATT G - 3′
	**AS:** 5′- CCA GTT TTT TTT TTT TTT TCA AAC ACC - 3′
**miR125a**	**S:** 5′ - AGT CCC TGA GAC CCT TTA AC - 3′
	**AS:** 5′ - GGT CCA GTT TTT TTT TTT TTT TCA CA - 3′
**miR125b**	**S:** 5′ - GCA GTC CCT GAG ACC CT - 3′
	**AS:** 5′ - CCA GTT TTT TTT TTT TTT TCA CAA GT - 3′
**miR126_3p**	**S:** 5′ - GCA GTC GTA CCG TGA GTA AT - 3′
	**AS:** 5′ - TCC AGT TTT TTT TTT TTT TTC GCA - 3′
**miR128_3p**	**S:** 5′ - CAC AGT GAA CCG GTC TC - 3′
	**AS:** 5′ - CAG GTC CAG TTT TTT TTT TTT TTT AAA G - 3′
**miR148_3p**	**S:** 5′ - GCA GTC AGT GCA TCA CAG A - 3′
	**AS:** 5′ - GGT CCA GTT TTT TTT TTT TTT TAC AAA - 3′
**miR181b**	**S:** 5′ - GCA GAA CAT TCA TTG CTG TC - 3′
	**AS:** 5′ - GTC CAG TTT TTT TTT TTT TTT ACC CA - 3′
**miR182**	**S:** 5′ - GTT TGG CAA TGG TAG AAC TCA - 3′
	**AS:** 5′ - GGT CCA GTT TTT TTT TTT TTT TAG TGT - 3′
**miR185**	**S:** 5′ - CAG TGG AGA GAA AGG CAG T - 3′
	**AS:** 5′ - GTC CAG TTT TTT TTT TTT TTT CAG GA - 3′
**miR191**	**S:** 5′ - CAA CGG AAT CCC AAA AGC A - 3′
	**AS:** 5′ - TCC AGT TTT TTT TTT TTT TTC AGC T - 3′
**miR192**	**S:** 5′ - CAG CTG ACC TAT GAA TTG ACA - 3′
	**AS:** 5′ - TCC AGT TTT TTT TTT TTT TTG GCT - 3′
**miR194**	**S:** 5′ - CAG TGT AAC AGC AAC TCC A - 3′
	**AS:** 5′ - TCC AGT TTT TTT TTT TTT TTC CAC AT - 3′
**miR195**	**S:** 5′ - GCA GTA GCA GCA CAG AAA TAT - 3′
	**AS:** 5′ - TCC AGT TTT TTT TTT TTT TTG CC - 3′
**miR200a**	**S:** 5′ - CAG TAA CAC TGT CTG GTA ACG - 3′

	**AS:** 5’ - GGT CCA GTT TTT TTT TTT TTT TAC ATC - 3’
**miR200b**	**S:** 5’ - CGC AGT AAT ACT GCC TGG T - 3’
	**AS:** 5’ - GGT CCA GTT TTT TTT TTT TTT TCA TCA - 3’
**miR200c**	**S:** 5’ - AGT AAT ACT GCC GGG TAA TGA - 3’
	**AS:** 5’ - GGT CCA GTT TTT TTT TTT TTT TCC A - 3’
**miR210_3p**	**S:** 5’- GCT GTG CGT GTG ACA - 3’
	**AS:** 5’- GTT TTT TTT TTT TTT TCA GCC GCT - 3’
**miR221_3p**	**S:** 5’- GCA GAG CTA CAT TGT CTG CT - 3’
	**AS:** 5’- CAG TTT TTT TTT TTT TTT GAA ACC CA- 3’
**miR222**	**S:** 5’ - GCA GAG CTA CAT CTG GCT - 3’
	**AS:** 5’ - CCA GTT TTT TTT TTT TTT TAC CCA GT - 3’
**miR223**	**S:** 5’ - CGC AGT GTC AGT TTG TC - 3’
	**AS:** 5’ - CCA GTT TTT TTT TTT TTT TGG GGT A - 3’
**miR301a_3p**	**S:** 5’ - CAG CAG TGC AAT AGT ATT GTC A - 3’
	**AS:** 5’ - GTC CAG TTT TTT TTT TTT TTT GCT T - 3’
**miR424_3p**	**S:** 5’ - CAA AAC GTG AGG CGC T - 3’
	**AS:** 5’ - GGT CCA GTT TTT TTT TTT TTT TAT AGC A - 3’
**miR451a**	**S:** 5’- CGC AGA AAC CGT TAC CAT TAC - 3’
	**AS:** 5’ - GGT CCA GTT TTT TTT TTT TTT TAA CTC - 3’

miRNA Primer; S: Sense; AS: Antisense
